# Child and adolescent mortality associated with pesticide toxicity in Cape Town, South Africa, 2010–2019: a retrospective case review

**DOI:** 10.1186/s12889-023-15652-5

**Published:** 2023-04-28

**Authors:** Bronwen Davies, Marie Belle Kathrina Mendoza Hlela, Hanna-Andrea Rother

**Affiliations:** 1 Division of Forensic Medicine and Toxicology, Department of Pathology, Faculty of Health Sciences, University of Cape Town, Anzio Road, Observatory, Cape Town, 7925 South Africa; 2 Forensic Toxicology Unit, Forensic Pathology Service, Western Cape Department of Health, Cape Town, South Africa; 3 Division of Environmental Health, School of Public Health, Faculty of Health Sciences, University of Cape Town, Anzio Road, Observatory, Cape Town, 7925 South Africa

**Keywords:** Adolescent pesticide suicides, Cape Town, Child pesticide poisoning, Paediatric, Mortality, Organophosphates, Street pesticides, South Africa, Terbufos

## Abstract

**Background:**

Poisoning of children after exposure to pesticides is a major public health concern, particularly in countries with poorer urban populations, such as South Africa. This may stem from the illegal distribution and domestic use of street pesticides, which are highly hazardous agricultural pesticides. The aim of this study was to profile paediatric deaths due to acute pesticide poisoning in the west-metropole of Cape Town, South Africa; to identify whether the active ingredients were highly hazardous pesticides according to the FAO and WHO; and to inform policy and public health interventions to prevent future exposures and mortality.

**Methods:**

A retrospective and descriptive analysis of forensic post-mortem records (2010 to 2019) was conducted to identify cases of paediatric deaths (< 18 years old) in the west metropole of Cape Town, involving pesticide poisoning admitted to the Salt River mortuary (one out of 16 mortuaries in the Western Cape province). Demographic, circumstantial, autopsy, and toxicological information was captured. Descriptive statistics, together with chi-square tests, Fisher’s probability tests, and Mann–Whitney U tests were used to analyse the data.

**Results:**

In total, 54 paediatric pesticide deaths were identified, including 22 (40.7%) males and 32 (59.3%) females, out of 5,181 paediatric unnatural deaths admitted over the 10-year period. The median age of the decedents was 8.3 years (range: 1 day to 17.9 years), with the majority under five years (42.6%) or between 15 and 18 years old (40.7%). All incidents occurred in peri-urban areas of Cape Town, with most individuals being admitted to hospital (88.9%) for a median survival time of 4.8 h. Toxicological analysis was requested in 50 cases (92.6%) with the organophosphate pesticides terbufos (*n* = 29), methamidophos (*n* = 2) and diazinon (*n* = 2) detected most frequently. Adolescent (15–18 years) suicides (29.6%) and accidental child deaths (< 4 years) (18.5%) were common.

**Conclusions:**

Terbufos and methamidophos are highly hazardous pesticide (HHP) active ingredients registered in South Africa for agricultural uses, yet commonly sold as street pesticides for domestic use in lower socioeconomic areas. Reducing access and availability of toxic pesticides, especially through the illegal selling of street pesticides, and providing low toxic alternatives to poorer communities, may support mortality reduction initiatives. Mortality and toxicology data provide important, often overlooked, surveillance tools for informing policy and public health interventions to reduce toxic pesticide harm in local communities.

## Background

Pesticides are commonly used in poor urban communities to control household pests where pest infestations are high [1]. The risk of pesticide poisoning to humans depends on the duration, frequency, and dose of the exposure, as well as the level of inherent toxicity of the pesticide. Human exposure—whether unintentional or intentional—typically occurs within occupational or domestic settings; through transfer from contaminated food, soil, air, or water; or through direct contact with the product. Recently, it was estimated that 385 million cases of accidental acute pesticide poisonings occur annually worldwide, resulting in approximately 11,000 *accidental* fatalities per year [2]. In terms of pesticide suicides, estimates range from 300,000 deaths [3, 4] to more recent conservative estimates of 110,000 deaths (13.7% of all global suicides) per year [5].

Children are uniquely vulnerable to pesticide exposure and toxicity [6–8]. In South Africa, paediatric cases of pesticide poisoning have been reported to be an increasing problem [9]. This may be because in many South African poor urban communities, residents search for cheap and effective methods to control domestic infestations of pests such as rats, cockroaches, flies, bedbugs, mosquitoes, etc. As a result, there is extensive sale and use of highly toxic pesticides sold illegally for domestic use, particularly in these lower socio-economic areas [1, 10, 11]. These toxic pesticides, known as street pesticides, are usually sold as liquids or granules in unlabelled containers, and are either pesticides registered only for agricultural use or unregistered products [1]. Street pesticides have been reported to contribute to the profile of paediatric poisoning in South Africa [1, 9, 11], resulting in approximately 11% of poisoning admissions to a Cape Town paediatric referral hospital [9, 12]. Local poison centres attributed pesticide poisoning to 16.7% of calls of infant poisoning [13], and 16.8% of calls for poisoning in children less than 13 years [14]. In the latter study, pesticide poisoning in children made up 40% of all pesticide poisoning calls [14]. While these studies allude to a few child deaths following hospital admission, there is currently no peer-reviewed published data (to the authors’ knowledge) that specifically investigates the profile of paediatric deaths due to pesticide toxicity in South Africa, and what percentage of these are linked to street pesticides.

In South Africa, pesticide poisoning and mortality is a notifiable medical condition (recorded as ‘poisoning agricultural stock remedies') [15]. Inadequate classification and underreporting of cases, together with ineffective surveillance systems, result in misinformed interventions and policy control [16]. In addition, in many poisoning cases—especially with street pesticides—the active ingredient is unknown or not reported, making it difficult to prevent risks from highly hazardous pesticides (HHPs). Poisonings are typically classified as ‘organophosphate poisoning’, or often not notified if other pesticides (e.g. carbamates, pyrethroids or coumarins) are involved, especially if symptoms are similar to other diseases and there is no history of poisoning [16]. Furthermore, incorrect conclusions about pesticides that cause poisoning can also be drawn based on popular belief or widespread misidentification of pesticides (e.g., identification of *any* dark granule as the pesticide aldicarb referred to colloquially as ‘two-step’), without formal toxicological tests.

Many of the street pesticides sold in South Africa are HHPs [1]. These are pesticides that are acutely or chronically hazardous to human health and/or the environment or listed on an international convention according to the Food and Agricultural Organization of the United Nations (FAO)/World Health Organisation (WHO) Joint Meeting on Pesticide Management (JMPM) criteria [17]. Recently, the WHO published updated recommendations for classifying pesticides by hazards and included pesticides that were deleted from previous versions because of continued use of the products particularly in low- and middle-income countries (LMICs) [18]. According to this document, previously reported street pesticides, including aldicarb and methamidophos, are classified as extremely and highly hazardous, respectively. Aldicarb is banned in South Africa due to its highly acute toxicity and its illegal use as a street pesticide [19]. What is needed in South Africa and other LMICs is up-to-date, consistent, and accurate surveillance data to prevent domestic use of HHPs.

Forensic mortality data provides an important and rich source of information to understand the causative agents of death [20], including paediatric deaths due to pesticides. Given the limited published data available on local pesticide fatalities, the aim of this study was to profile paediatric deaths due to pesticide poisoning in the west metropole of Cape Town, South Africa, and establish the relevance of using forensic data for pesticide surveillance. Furthermore, we aimed to identify the active ingredients involved in these deaths, determine whether any were HHPs according to the FAO and WHO, as well as to inform policy and public health interventions to prevent future exposures and mortality.

## Methods

### Setting

In South Africa, all suspected unnatural death cases are admitted to provincial Department of Health, Forensic Pathology Service (FPS) facilities, for post-mortem examinations by an authorized forensic pathologist, so as to determine the circumstances and causes of death [21, 22]. Pesticide-related deaths typically undergo a standard autopsy during which biological specimens—most commonly peripheral whole blood, urine, vitreous humor, and gastric contents—are collected for ancillary investigations such as toxicology. The forensic pathologist determines the cause of death using evidence from the scene investigation, the clinical history, their autopsy findings, and the ancillary test results.

Salt River Mortuary (SRM) is one of 16 FPS mortuaries in the Western Cape Province (WCP) of South Africa. The Salt River and Tygerberg mortuaries serve the Cape Town metropole and are the two busiest in the province, each receiving over 4,000 unnatural death admissions annually (of over 11,000 total unnatural death case admissions in the province) [23]. SRM serves the west metropole of the City of Cape Town, which encompasses the Western, Southern, Klipfontein, and Mitchells Plain districts containing a population of over 1,937,380. An estimated 621,628 (32.1%) of this population are 19 years old or younger. These population data were calculated for the SRM drainage area using data obtained from the City of Cape Town, Information and Knowledge Management Department, which was based on 2011 National Census data compiled by StatsSA [24].

### Study design

A retrospective, cross-sectional study design was adopted to identify paediatric cases (< 18 years of age) of acute fatal pesticide poisoning admitted to SRM between 1 January 2010 and 31 December 2019. The operational Office Autopsy Database (OAD), housed in the Division of Forensic Medicine and Toxicology at the University of Cape Town (UCT), was retrospectively reviewed to identify cases in which pesticide toxicity was suspected to have caused or contributed to the death of an individual. The database contains all information pertaining to all unnatural deaths at SRM, as documented by the forensic pathologists conducting the autopsies. Pathologists routinely record basic information for their cases, including the individual’s demographic information, the date of death and autopsy, the circumstances surrounding death, the cause and suspected manner of death, and whether ancillary testing or investigations were requested or performed following autopsy.

### Inclusion criteria

To be eligible for inclusion, decedents had to be under 18 years old, have died between January 2010 and December 2019 (inclusive), and have evidence or suspicion of pesticide poisoning (usually noted in the cause of death or circumstantial history) as indicated in the OAD. Any missing electronic cases (as denoted by missing information in the OAD), were followed up by review of the hard-copy post-mortem reports, to include or exclude as pesticide-related. Cases in which poisoning was suspected, but there was no indication in available documentation that it was pesticide-related, were excluded (e.g., herbal/traditional medicine or drugs). All cases preliminarily identified as paediatric pesticide deaths were confirmed through review of the post-mortem records.

### Data collection and analysis

All data were verified by reviewing the post-mortem records, which included the post-mortem report, scene investigation forms, summarised hospital forms related to the death and interventions (if they died in hospital), and blood alcohol and toxicology results. Data collected included demographic information (i.e., age, sex, location and time of incident and death); date and time of death (declaration) and autopsy; date and time of hospital admission, hospital interventions and clinical findings; location and area of incident; alleged manner and cause of death, and specific autopsy observations; and any toxicological investigations and results. Data were collated in Excel and analysed using the IBM© Statistical Package for Social Sciences (SPSS©) Statistics 25.0 (SPSS Inc., Chicago, IL). Descriptive statistics, together with statistical chi-square tests and Fisher’s probability tests were applied. Mann–Whitney U tests were used for non-parametric data. Statistical significance was accepted at *p* < 0.05.

### Toxicological analyses

Formal toxicological analyses were performed by the National Department of Health’s Forensic Chemistry Laboratory (FCL) in Woodstock, Cape Town. All pesticide analyses by FCL were qualitative and thus no quantitative concentration data were available. No volatile or surfactant analyses were performed in these cases despite the relevance of these to the toxicity of certain pesticide formulations. Pharmacological screening was performed by the UCT Division of Pharmacology. This was performed on a high-performance liquid chromatography, tandem mass spectrometry system (LC–MS/MS) until 2018, after which the screen was performed on an LC quadrupole time-of-flight (LC-QTOF-MS) system. Pathologists requested this screen on an ad-hoc basis while awaiting formal toxicology results. The screen did not, however, routinely include pesticides. Toxicological results were obtained, where available, from Pharmacology and FCL reports submitted to FPS. No analyses were performed by the authors.

### Ethics

Ethical approval to conduct and publish this research was granted by Human Research Ethics Committee (HREC) of UCT’s Health Sciences Faculty (HREC Ref: 592/2016). Consent from the next-of-kin was not required by the UCT HREC, as the study was a retrospective records review. In addition, the results reviewed were obtained from routine post-mortem investigations, in which consent was not required for the mandated collection and testing of post-mortem specimens for medico-legal investigations, as per South Africa’s Inquest Act (No. 58 of 1959). The OAD repository also has UCT HREC approval (HREC Ref: R016/2014).

## Results

### Paediatric deaths at Salt River Mortuary

**Fig. 1:**
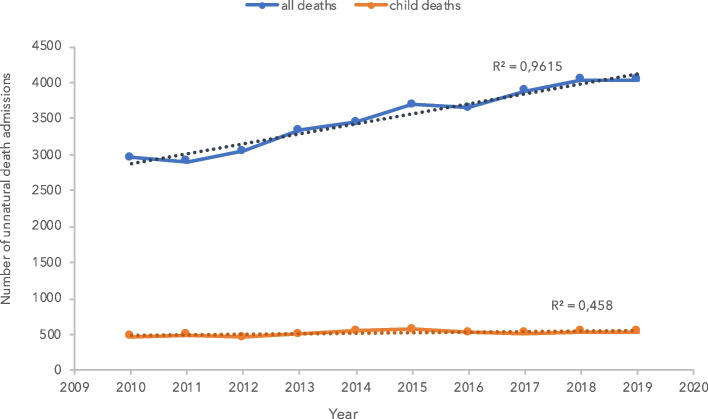
Number of all (blue) and paediatric (orange) unnatural death admissions to Salt River mortuary, 2010–2019

The number of all-cause suspected unnatural deaths admitted to SRM increased steadily from below 3,000 to greater than 4,000 cases from 2010 to 2019, respectively (Fig. 1). Of these, there were a total of 5,181 paediatric unnatural deaths over this 10-year period. In the latter five years, the number of paediatric unnatural deaths averaged 543 (SD ± 22) cases annually. This accounted for approximately 13–16% of all suspected unnatural death admissions in each year.

### Profile of paediatric pesticide deaths

In total, 54 paediatric pesticide-related deaths were admitted to SRM between 2010 and 2019. These deaths were not evenly distributed over the ten-year period, with an average of five cases (SD ± 3) occurring per year (accounting for < 1% of unnatural paediatric death admissions). Of these 54 fatalities, the majority (*n* = 32; 59.3%) were female (Table 1). The sample population varied greatly in age with a median of 8.3 years, ranging from 1 day to 17.9 years old. Most deaths involved adolescents aged 15 years or older (*n* = 22, 40.7%), younger children under 5 years old (*n* = 16, 29.6%), and infants (< 1 year old) (*n* = 7, 13.0%). Older children over 15 years of age were primarily mainly females (4.5:1 ratio), while the younger individuals (1–4 years old) were predominantly males (*p* = 0.01). For the remaining age groups, there was equal distribution between the two sexes.

**Table 1:** Characteristics of paediatric pesticide-related fatalities (*N* = 54) in the west metropole of Cape Town, 2010–2019

Number of paediatric pesticide-related deaths, n (%)							
**Age (years)**	** < 1** 7 (13.0)	**1–4** 16 (29.6)	**5–9** 5 (9.3)	**10–14** 4 (7.4)	** ≥ 15** 22 (40.7)	**total** 54 (100.0)	***p*-value**
**Biological sex**							0.031
Female	3 (42.9)	6 (37.5)	2 (40.0)	3 (75.0)	18 (81.8)	32 (59.3)	
Male	4 (57.1)	10 (62.5)	3 (60.0)	1 (25.0)	4 (18.2)	22 (40.7)	
**Alleged manner of death**							< 0.001
Homicide	2 (28.6)	2 (12.5)	0 (0.0)	1 (25.0)	0 (0.0)	5 (9.3)	
Suicide	0 (0.0)	0 (0.0)	0 (0.0)	2 (50.0)	16 (72.7)	18 (33.3)	
Accident	1 (14.3)	9 (56.2)	2 (40.0)	0 (0.0)	1 (4.5)	13 (24.1)	
Under investigation	4 (57.1)	5 (31.3)	3 (60.0)	1 (25.0)	5 (22.7)	18 (33.3)	

Overall, the most common alleged manner of deaths (at the time of autopsy) were suicides (*n* = 18, 33.3%), and accidents (*n* = 13, 24.1%), with 33% of the cases recorded as under investigation following autopsy, pending ancillary investigations such as toxicology. Most suicide cases involved adolescents (≥ 15 years) (*n* = 16, 72.7%), whereas accidental deaths involved younger children (1–4 years) (*p* < 0.001). There were five cases of suspected homicides, which involved the deceased being fed pesticides by a parent or caregiver (*n* = 3), or by strangers (after the deceased had exposed them for stealing) (*n* = 1). The final homicide case involved an abandoned newly born infant with pesticides in their system (*n* = 1).

The poisoning incidents were all acute exposures in residential settings, which typically occurred within lower socioeconomic areas within the greater Cape Town area. These included Gugulethu (*n* = 21, 38.9%), Mitchell’s Plain (*n* = 8, 14.8%), Philippi (*n* = 6, 11.1%), Nyanga and Browns Farm (*n* = 4, 7.4%), Samora Machel (*n* = 3, 5.6%); as well as Philippi East, Khayelitsha, Langa and Crossroads (*n* = 2, 3.7% each). These are all peri-urban areas that contain dense informal settlements. Although some of these areas fell outside of the west metropole of Cape Town (e.g., Khayelitsha), these individuals were transferred to hospitals within the SRM drainage area before they died.

Most of the individuals (*n* = 48, 88.9%) were admitted to hospital, however; of those admissions, 17 cases (31.5%) were classified as dead-on-arrival (DOA). For those who survived for a longer period in hospital (*n* = 31, 57.4%), the duration of hospitalisation varied widely between nine minutes and 11 days (median: 4.8 h). Most individuals were admitted to hospital or died on scene during the evening (18h00-00:00; 46.3%) or afternoon (12h00-18h00; 25.9%) on Mondays, Wednesdays, and Thursdays, and in the months of March, April and October. However, no statistical significance was observed between the time, day, month, or year of the initial poisoning incident itself. The clinical presentation and recorded history of the 54 fatalities varied widely depending on whether the individual was hospitalised and managed by a clinician or whether they died at home. A combination of the observations recorded by scene investigators, clinicians, or pathologists are presented in Table 2.

**Table 2:** Key observations recorded in the paediatric pesticide-related fatalities (*N* = 54)

Observations	No. of cases n (% of total)
**Incident or scene history**	
History of recently eating/drinking	15 (27.8)
Pesticide found near deceased	4 (7.4)
Patient ingested *milk* to ‘treat’ poisoning^a^	4 (7.4)
**Exposure/poisoning**	
Suspected poison ingestion^b^	46 (85.2)
Clinician suspected pesticide toxicity	25 (46.3)
**Hospital treatment**	
Medical intervention	38 (70.4)
Atropine administered	27 (50.0)
**Recorded signs or symptoms**	
Unresponsive	41 (75.9)
Pin-point pupils	27 (50.0)
Shortness of breath/gasping	23 (42.6)
Salivating/secretions	21 (38.9)
Fasciculations/seizures	19 (35.2)
Vomiting	16 (29.6)
Foam around mouth	15 (27.8)
Diarrhea/bowel movements	8 (14.8)
Bradycardia	7 (13.0)
Cardiac/respiratory arrest	5 (9.3)
Stomach pain	4 (7.4)
Sweating	2 (3.7)
Dizziness	1 (1.9)
**Laboratory testing**	
Low pseudocholinesterase^c^	15 (27.8)
**Autopsy findings**	
Granules^d^ noted in gastric content	19 (35.2)
Congested organs	5 (9.3)

^a^There remains a common misconception in South African communities that milk should be used to treat poisoning

^b^Specific reference made to ‘rattex’ (5), rat poison (6), OP (1), weed killer (1), garden poison (1), flea dip (1)

^c^Four pseudocholinesterase results were obtained from post-mortem whole blood specimens

^d^Black pepper/millet seed-like (10), grey (7), pale blue (1), colour unspecified (1)

Observations were neither routinely recorded nor standardised. As a result, the lack of observation does not exclude that factor in the case. Likewise, observations were not mutually exclusive. *Rattex*, a commercially available pesticide commonly sold as pink pellets containing brodifacoum, was reported in at least five cases. In three of these cases, the gastric content at autopsy actually contained grey or black granules, suggesting a street pesticide.

### Autopsy and toxicological findings

The mean post-mortem interval (PMI) period for the 54 deaths was 3.7 days (range: 1–11 days). The most common reported observations at autopsy included non-specific signs of organ congestion and froth in the airways, as well as more specific findings of pungent smells or distinct granules (i.e., black, grey, or blue) present in the decedent’s gastric content (Table 2). In half (*n* = 27) of the cases, the cause of death was reported as consistent with pesticide (usually organophosphate) poisoning or ingestion (Table 3). In five (9.3%) cases, the pathologist recorded the same conclusion, but also indicated that the final cause of death was pending toxicological results. For the remaining cases (*n* = 22, 40.7%), the initial cause of death was reported as undetermined by autopsy alone and remained under investigation. These cases were typically characterised by a history of a toxic ingestion, and the absence of relevant pathology at autopsy, apart from granules in the gastric content or pesticides reported on scene. However, the pathologists would wait for toxicological results to subsequently release a final cause of death report at a later stage. While most of these toxicological reports were released at the time of the study, the final cause of death report was not necessarily available at the time of the study.

**Table 3:** Cause of death of the paediatric pesticide fatalities according to manner of death and toxicological results

Number of paediatric pesticide-related deaths, n (%)				
**Cause of death**	**Consistent with OP**^**a**^** poisoning or ingestion** 27 (50.0)	**Consistent with OP**^**a**^** poisoning or ingestion but awaiting toxicology** 5 (9.3)	**Under investigation**^**c**^ 22 (40.7)	**Total** 54 (100.0)
**Manner of death**				
Homicide	3 (11.1)	0 (0.0)	2 (9.1)	5 (9.3)
Suicide	9 (33.3)	2 (40.0)	7 (31.8)	18 (33.3)
Accident	8 (29.6)	2 (40.0)	3 (13.6)	13 (24.1)
Under investigation	7 (25.9)	1 (20.0)	10 (45.5)	18 (33.3)
**Pharmacology screening**				
No	13 (48.1)	3 (60.0)	14 (63.6)	30 (55.6)
Yes	14 (51.9)	2 (40.0)	8 (36.4)	24 (44.4)
**Formal toxicological analysis**				
No	4 (14.8)	0 (0.0)	0 (0.0)	4 (7.4)^b^
Yes	23 (85.2)	5 (100.0)	22 (100.0)	50 (92.6)

^a^*OP* Organophosphate

^b^All four cases had a long survival period at hospital (16, 79, 96, and 247 h)

^c^Autopsy reports were released as preliminary, while the pathologist was waiting for toxicological results to determine the final cause of death. Final cause of death reports were not necessarily available at the time of the study

A pharmacology drug screen was performed in 44.4% of the cases, while formal forensic toxicological analyses were requested in 92.6% of the cases (Tables 3 and 4). The pharmacology screen did not typically include analysis for pesticides and organophosphate metabolites; however, this was performed on an ad hoc basis illustrated by the few pesticide detections (*n* = 6) (Table 4).

**Table 4:** Toxicological findings for the suspected pesticide poisoning cases according to testing method

Toxicological results	No. of cases n (% of analysed cases)
**Pharmacology screening (*N* = 24)**	
No drugs detected	12 (50.0)
Drugs^a^	4 (16.7)
Organophosphate pesticide metabolites (diethyl thiophosphate/diethyl dithiophosphate)	3 (12.5)
Atropine only	2 (8.3)
Terbufos (trace) + drugs^b^	2 (8.3)
Carbendazim	1 (4.2)
**Formal toxicological analysis (*N* = 50)**	
Terbufos	26 (52.0)
Outstanding	9 (18.0)
Drugs^c^	6 (1.2)
Terbufos + drugs^d^	2 (4.0)
Methamidophos	2 (4.0)
Diazinon	2 (4.0)
Carbofuran + drugs^e^	1 (2.0)
Phorate + terbufos + ethion	1 (2.0)
No drugs or pesticides detected	1 (2.0)
**Blood alcohol concentration (BAC) analysis (*N* = 12)**	
BAC < 0.01 g/100 mL (not detected)	11 (91.7)
BAC = 0.02 g/100 mL	1 (8.3)

^a^1: acetaminophen, diazepam, lidocaine, midazolam; 2: acetaminophen, salicylamide, methamphetamine, diphenhydramine; 3: phenytoin; 4: amidarone, hyoscamine, diclofenac

^b^1: atropine; 2: metoclopramide, morphine;

^c^1:midazolam, orphenadrine, phenobarbital; 2: diphenhydramine, doxylamine; 3: chlorpheniramine, orphenadrine; 4: methamphetamine, morphine; 5: acetaminophen; 6: isoniazid

^d^1: acetaminophen; 2: metoclopramide, morphine

^e^1: acetaminophen

At the time of this study conclusion, 18.0% of laboratory test results were still outstanding from the National FCL. From the results received, the organophosphate terbufos was the most common pesticide reported (58.0%) (Table 4). In two of these cases, terbufos was found in combination with other drugs, and in one other case, it was reported alongside the organophosphate pesticides phorate and ethion. Methamidophos and diazinon (both organophosphates) were separately detected in two cases each, while carbofuran (carbamate) was identified in one case only. Blood was collected for ethanol (alcohol) analysis (BAC) in 12 cases (20.5%). For these cases, all except two decedents (8.7 and 12.5 years) were above the age of 15 years. Ethanol was not detected (< 0.01 g/100 mL) in 11 of the 12 cases (91.7%). In one case it was just above the limit of detection at 0.02 g/100 mL.

For the four cases in which formal toxicology was not requested, the individuals were in hospital for 16 h, and 3, 4, and 10 days respectively. In these cases, there was a history of pesticide or ‘rat poison’ ingestion, the clinical toxidrome suggested organophosphate or carbamate toxicity and ante-mortem pseudocholinesterase levels were low. The forensic pathologists thus determined the cause of death to be consistent with organophosphate poisoning in all cases.

## Discussion

Pesticide poisoning in children has been reported to be an increasing problem in South Africa [9, 12]; however, there is little published data on *fatal* poisonings, with clinical case reports stemming from hospital and Poison Information Centre (PIC) records [9, 12–14, 25, 26]. This data is particularly important for evidence-based decision-making by pesticide regulators. This study investigated paediatric unnatural deaths and found that while the unnatural death admissions to SRM continued to rise each year, paediatric deaths in the west metropole of Cape Town remained constant over the investigated period. Acute pesticide poisoning accounted for only a small percentage (< 1%) of these fatalities. This is likely an underestimate, particularly due to the delayed national toxicological services in the country during the study period, upon both which the cause of death and the analytical identification of a pesticide, were reliant.

Children are vulnerable to pesticide exposure because of their behavioural patterns and developing physiology, and they may not be able to detoxify chemical exposures as adults can (i.e., their windows of vulnerability) [27, 28]. Children under five years in this cohort were noted to be particularly at risk of accidental and homicidal poisonings, aligning with findings in a local clinical setting [9] and Child Death Reviews (CDR) [29, 30]. In poor communities with low quality housing, children often have easy access to highly hazardous pesticides either because there are no storage facilities or because they are accessible after being mixed with food for pest control [1]. A high number of adolescent suicides, particularly amongst females over 15 years, was also identified in this study. The prevalence of suicide has been noted to increase during adolescence [29, 30], as stressors (e.g., social pressures) and mental disorders develop [31], and poisoning is a common method of suicide for women [32].

Toxic profiles may also be related to socioeconomic status [33], and financial hardship and other domestic challenges are notable underlying risk factors for self-harm [29, 33]. Pesticide poisonings were previously reported within poorer peri-urban areas in Western Cape [9, 12], similar to the locations of the poisoning incidents in this study (e.g. Guglethu, Mitchell’s Plain and Nyanga). In these areas, highly toxic street pesticides are used to control domestic pests, are widely available to purchase, and are in easy reach of children in the home, resulting in accidental or intentional ingestions [1, 11]. Most of the included paediatric cases were hospitalised before death, with signs of cholinergic toxicity being frequently recorded by clinicians. Children may, however, present with varying symptoms of toxicity to adults, which can complicate diagnoses [34, 35]. Cholinesterase inhibitors (e.g., organophosphates and carbamates) have previously been reported to cause local poisonings according to clinical toxidromes and/or other history of ingestion [9, 12–14, 25, 26]. Together with other reports of visual identification of the pesticide related to poisonings [36], these studies allude to the troubling role of toxic street pesticides in poisonings in South Africa.

Street pesticides are highly hazardous pesticides (HHPs) that contain toxic agricultural pesticides that are sold unlabelled as decanted liquids or granules in an illegal manner, with no warnings or instructions for use [1]. The highly acutely neurotoxic carbamate aldicarb, was previously noted as a street pesticide that played a prominent role in poisoning cases [1, 9, 14]. Colloquially known as ‘rat poison’, ‘rattex’ and ‘two-step’, aldicarb was sold by informal traders in the form of a straw of black granules [14]. While aldicarb may still be involved in poisonings, this study highlighted the role of the toxic organophosphate terbufos in pesticide fatalities. In five cases where ‘rattex’ was recorded by investigators, terbufos was detected in three of those cases, with the other two cases outstanding toxicology or not having pesticides detected. Where pathologists reported identification of ‘aldicarb black granules’ in gastric content (*n* = 8), no aldicarb was reported as detected, and most of these analytically confirmed as terbufos (*n* = 5). Our hypothesis is that after aldicarb was voluntarily withdrawn from the industry in 2013 and prohibited for sale and use in agriculture by the South African Department of Agriculture in 2016 [29], terbufos, which is still legally sold for similar uses in agriculture, was the street pesticide replacement for rat control. Terbufos is formulated as small grey granules and may be mistaken as aldicarb to the naked eye. This study illustrates the importance of analytical toxicology in monitoring of pesticides involved in poisoning, which in this case point to terbufos and not aldicarb causing child deaths.

Terbufos is classified by the WHO as an extremely hazardous (class Ia) pesticide [18]. Other minor pesticides involved in deaths in this study included extremely hazardous (Ia) phorate, highly hazardous (Ib) methamidophos and carbofuran, and moderately hazardous (II) diazinon and ethion [18]. These are pesticides that have no residential use; however, all poisoning incidents in this study occurred in domestic settings. Terbufos is available at local cooperatives within the Western Cape province of South Africa. A license is not required to purchase terbufos, and therefore anyone wishing to sell it as a street pesticide in the informal sector can do so. Only a few global case reports of terbufos poisoning fatalities have been published [37, 38] relating to occupational contact or availability of the pesticide. To the authors’ knowledge, this is the first publication that illustrates that terbufos is linked to paediatric poisonings and deaths in South Africa.

Despite hospitalisation and delayed death, post-mortem specimens were collected for formal toxicological analyses in most cases. Longer survival reduces the likelihood of detecting pesticide analytes due to metabolism, elimination, and hospital interventions. In six cases, only drugs detections were reported (no pesticides), two of which had ‘black’ granules recorded in the gastric content. This suggests that testing may not have been adequate to identify the relevant pesticide. In two more of these cases, only blood and urine were submitted for toxicology. The laboratory did not analyse for pesticide metabolites, which is relevant for both specimens, and all blood samples were collected in vials of 2–3% sodium fluoride (preservative), which may enhance degradation of organophosphate pesticides [39]. In addition, testing was delayed from months to years in most cases, and the likelihood of degradation in blood was high. In fact, in most cases, pesticides were qualitatively detected only in the gastric content, which forensic pathologists deemed sufficient to determine the cause of death, often in combination with clinical presentation before death.

In almost 20% of the cases, the toxicology results were still outstanding, due to backlogs in the national FCL laboratories. This not only impacts the conclusion of many inquest or criminal court cases, but also limits the relevance and up-to-date use of toxicological data for public health interventions. To combat these systemic challenges, the FPS Forensic Toxicology Unit (FTU) has developed an internal toxicology service within FPS that aims to reduce the turn-around of post-mortem toxicology in the province, provide integrated analytical and interpretive toxicological support, and be actively involved in public health initiatives using toxicological data.

Prevention of child and adolescent pesticide deaths.

What is clear from this study is that access to HHPs, including street pesticides, continues to promote accidental ingestion by children and intentional use by adolescents. As has been shown in other studies, the banning of HHPs and registering of low risk pesticides play a significant role in the reduction of pesticide related deaths [40–49]]. Although pesticide poisoning is a notifiable condition in South Africa, the current reporting system is failing to inform pesticide policy and regulation. Enhancing and standardising clinical and/or post-mortem registries, and integrating these with high-quality, comprehensive, and timeous analytical toxicology data, is essential for successful toxicosurveillance [50]. The development and capacitating of new public forensic toxicology laboratories, such as the FTU within FPS in WCP, may demonstrate the local government’s agenda to improve systems for child safety and death prevention. We, therefore, advocate for rigorous systems to be put in place in South Africa (and other countries) for enhancing support for routine toxicological analyses and the subsequent utilisation of pesticide mortality data by policy makers in a timely and systematic manner. Child pesticide deaths and adolescent pesticide suicides need to be urgently addressed to prevent future deaths.

Study limitations.

This study provided a snapshot of approximately one third of unnatural deaths from one of 16 mortuary facilities in the Western Cape province and is not generalisable to the South African paediatric population. The authors recommend that further research be conducted to include pesticide mortuary data from all mortuaries within South Africa. It is recognised that there are limitations with the use of a secondary dataset to identify cases, especially given the inconsistent recording of data by pathologists (e.g. clinical presentation, scene investigation, and cause of death). Implementing a provincial protocol for FPS forensic officers at the scene or hospital investigation of these cases, would improve the quality of routine data collected, as would the standardisation of cause of death determinations.

The researchers highlight the limitation of not having access to comprehensive and timeous toxicological results for post-mortem cases. This has been a historically long-standing issue within South Africa and may result in an underestimation of cases where toxicological results may provide the only probative information to determine whether pesticides were involved in causing death. In addition, only qualitative results were available, and the comprehensive and reliable nature of testing was not known to the authors. The analysis of toxic volatiles and surfactants present in some pesticide formulations would be important in the future. Finally, this study did not assess the number of incidents in which the statutory requirement of notification of pesticide poisoning to the local health authority was met. It is recognised that notification is problematic and may result in under-reporting and inaccurate national statistics [16].

## Conclusions

This article provides insight into paediatric pesticide poisoning fatalities admitted to one of the busiest mortuaries in Cape Town, South Africa. While pesticides cause a minor number of paediatric deaths in the mortuary data reviewed, what is key is that these are preventable and could be occurring on a much larger scale if all mortality data was reviewed. Terbufos was most frequently involved in deaths, stemming from its distribution and domestic use as a street pesticide and policies controlling its availability and use should be implemented. While National toxicology data was lacking, this study illustrated the importance of forensic mortality and toxicological data in pesticide surveillance used for regulating pesticides in South Africa and globally.

## Data Availability

The datasets generated and/or analysed during the current study are not openly available due to its sensitive and medico-legal nature. The data are available from the corresponding author on reasonable request.

## Abbreviations

BAC: Blood alcohol concentration
DOA: Dead-on-arrival
FAO: Food and Agricultural Organization of the United Nations
FCL: Forensic Chemistry Laboratory
FPS: Forensic Pathology Service
FTU: Forensic Toxicology Unit
GHS: Globally Harmonized System of Classification and Labelling of Chemicals
HHPs: Highly hazardous pesticides
HREC: Human Research Ethics Committee
LC–MS/MS: Liquid chromatography, tandem mass spectrometry
LC-QTOFMS: Liquid chromatography quadrupole time-of-flight mass spectrometry
LMICs: Low- and middle-income countries
OAD: Office autopsy database
OP: Organophosphate
PIC: Poison Information Centre
PMI: Post-mortem interval
SAMRC: South African Medical Research Council
SD: Standard deviation
SPSS: Statistical Package for Social Sciences
SRM: Salt River mortuary
UCT: University of Cape Town
WHO: World Health Organization
